# Can postoperative mean transprosthetic pressure gradient predict survival after aortic valve replacement?

**DOI:** 10.1007/s00392-013-0629-3

**Published:** 2013-10-18

**Authors:** Bart M. Koene, Mohamed A. Soliman Hamad, Wobbe Bouma, Massimo A. Mariani, Kathinka C. Peels, Jan-Melle van Dantzig, Albert H. van Straten

**Affiliations:** 1 Department of Cardiothoracic Surgery, Catharina Hospital, Michelangelolaan 2, Postbus 1350, 5602 ZA Eindhoven, The Netherlands; 2 Department of Cardiothoracic Surgery, University Medical Center Groningen, Hanzeplein 1, Postbus 30.001, 9700 RB Groningen, The Netherlands; 3 Department of Cardiology, Catharina Hospital, Michelangelolaan 2, Postbus 1350, 5602 ZA Eindhoven, The Netherlands

**Keywords:** Prosthesis, Mismatch, Aortic valve, Replacement, Gradient, Survival analysis

## Abstract

**Background:**

In this study, we sought to determine the effect of the mean transprosthetic pressure gradient (TPG), measured at 6 weeks after aortic valve replacement (AVR) or AVR with coronary artery bypass grafting (CABG) on late all-cause mortality.

**Methods:**

Between January 1998 and March 2012, 2,276 patients (mean age 68 ± 11 years) underwent TPG analysis at 6 weeks after AVR (*n* = 1,318) or AVR with CABG (*n* = 958) at a single institution. Mean TPG was 11.6 ± 7.8 mmHg and median TPG 11 mmHg. Based on the TPG, the patients were split into three groups: patients with a low TPG (<10 mmHg), patients with a medium TPG (10–19 mmHg) and patients with a high TPG (≥20 mmHg). Cox proportional-hazard regression analysis was used to determine univariate predictors and multivariate independent predictors of late mortality.

**Results:**

Overall survival for the entire group at 1, 3, 5, and 10 years was 97, 93, 87 and 67 %, respectively. There was no significant difference in long-term survival between patients with a low, medium or high TPG (*p* = 0.258). Independent predictors of late mortality included age, diabetes, peripheral vascular disease, renal dysfunction, chronic obstructive pulmonary disease, a history of a cerebrovascular accident and cardiopulmonary bypass time. Prosthesis–patient mismatch (PPM), severe PPM and TPG measured at 6 weeks postoperatively were not significantly associated with late mortality.

**Conclusions:**

TPG measured at 6 weeks after AVR or AVR with CABG is not an independent predictor of all-cause late mortality and there is no significant difference in long-term survival between patients with a low, medium or high TPG.

## Background

Implantation of a prosthetic aortic valve too small for the patient’s body size could lead to an increased hemodynamic burden by creating left ventricular outflow obstruction, resulting in a higher mean transprosthetic pressure gradient (TPG). This condition after aortic valve replacement (AVR) is known as prosthesis–patient mismatch (PPM) and occurs when the effective orifice area (EOA) of the implanted valve prosthesis is too small in relation to the body surface area (BSA) of the patient [1, 2]. PPM is expressed by the indexed EOA (EOAI). The EOAI is calculated by dividing the corresponding EOA of each valve type and size by each patient’s BSA. Although different cut-off values exist to define PPM, usually a cut-off value of EOAI ≤0.85 cm^2^/m^2^ is chosen, as described by Pibarot and colleagues [1]. An EOAI <0.65 cm^2^/m^2^ is regarded as severe PPM [1, 3, 4]. The EOAI has been shown to negatively correlate with the TPG [5–7] and other studies have shown that despite normal prosthesis function, relatively high TPG can be measured after AVR [3, 4, 8–12].

Studies examining the impact of an undersized prosthetic aortic valve on long-term survival mainly focus on describing the existence of PPM. The impact of PPM on mortality after AVR is still a controversial topic. Several studies have shown that PPM is associated with increased short-term and/or long-term mortality after AVR [13–16]. Other studies contradict these findings and report that PPM does not have a significant impact on survival [17–24].

In this study we focussed on the main hemodynamic consequence of PPM, and we sought to determine the effect of a higher TPG, measured at 6 weeks after AVR or AVR with coronary artery bypass grafting (AVR with CABG), on late all-cause mortality.

## Methods

### Study design

This is a retrospective, observational study on consecutive patients. Data were obtained from the Institutional database, normally utilized for patient care. Clinical data, echocardiographic data, catheterization data, and surgical reports were entered into the institutional database prospectively and analyzed retrospectively. Because anonymous standard clinical follow-up check-ups were used to collect and analyze data, the study was approved by the Medical Ethical Committee.

### Patients

Between January 1998 and March 2012, 2,957 patients underwent AVR (*n* = 1,701) or AVR with CABG (*n* = 1,256) using a mechanical or stented biological aortic valve prosthesis at our institution. Only patients who underwent transthoracic echocardiography (TTE) at 6 weeks after AVR or AVR with CABG were analyzed, leading to the exclusion of 681 patients, including 57 patients who died within 6 weeks postoperatively (early mortality < 6 weeks = 1.9 %). Twelve patients were lost to follow-up and were also excluded from our analysis.

Based on the TPG the patients were split into three groups: patients with a low TPG (<10 mmHg), *n* = 876; patients with a medium TPG (10–19 mmHg), *n* = 1,184; and patients with a high TPG (≥20 mmHg), *n* = 204. Patient characteristics are summarized in Table 1.

**Table 1:** Characteristics for each pressure gradient group (*n* = 2,264)

Variable	Low gradient	Moderate gradient	High gradient	*p*
	(<10 mmHg) *n* = 876	(10–19 mmHg) *n* = 1,184	(≥20 mmHg) *n* = 204	
Age (years)	70 ± 10	67 ± 11	65 ± 12	<0.001
Sex				
Female	328 (37.4)	448 (37.8)	56 (27.5)	0.015
Endocarditis	27 (3.1)	54 (4.6)	14 (6.9)	0.035
Preoperative LV function				
Severely impaired (EF < 30 %)	39 (4.5)	27 (2.3)	4 (2.0)	0.012
Hypertension	406 (46.3)	549 (46.4)	78 (38.2)	0.085
Diabetes mellitus	151 (17.2)	203 (17.1)	33 (16.2)	0.934
Body weight (kg)	77 ± 13	79 ± 14	81 ± 16	<0.001
Height (cm)	170 ± 9	170 ± 9	171 ± 9	0.172
Body surface area, BSA (m^2^)	1.88 ± 0.19	1.91 ± 0.19	1.92 ± 0.21	0.002
Body mass index, BMI (kg/m^2^)	26.8 ± 4.0	27.4 ± 4.2	27.5 ± 4.7	0.006
Peripheral vascular disease (PVD)	108 (12.3)	111 (9.4)	20 (9.8)	0.091
Renal dysfunction	46 (5.3)	65 (5.5)	11 (5.4)	0.972
Chronic obstructive pulmonary disease (COPD)	144 (16.4)	194 (16.4)	41 (20.1)	0.404
Cerebrovascular accident (CVA)	36 (4.1)	64 (5.4)	12 (5.9)	0.305
Previous cardiac surgery	64 (7.3)	85 (7.2)	31 (15.2)	<0.001
Additive EuroSCORE	6.2 ± 2.5	5.9 ± 2.5	5.8 ± 2.5	0.052
Logistic EuroSCORE	7.93 ± 8.33	7.06 ± 7.06	7.18 ± 7.69	0.075
Prosthetic valve diameter, mm				
Median	23	23	23	
Prosthetic valve type				
Mechanical	367 (41.9)	580 (52.3)	120 (58.8)	<0.001
Concomitant coronary artery bypass grafting	424 (48.4)	464 (39.2)	66 (32.4)	<0.001
Cardiopulmonary bypass time (min)	95 ± 37	91 ± 36	93 ± 32	0.024
Aortic cross-clamp time (min)	70 ± 26	68 ± 25	68 ± 22	0.147
Effective orifice area (EOA) (cm^2^)	2.16 ± 0.48	2.06 ± 0.42	1.94 ± 0.40	<0.001
Indexed effective orifice area (EOAI) (cm^2^/m^2^)	1.15 ± 0.24	1.08 ± 0.21	1.01 ± 0.21	<0.001
PPM (EOAI ≤0.85 cm^2^/m^2^)	43 (4.9)	145 (12.2)	51 (25.0)	<0.001
Severe PPM (EOAI <0.65 cm^2^/m^2^)	2 (0.2)	2 (0.2)	3 (1.5)	0.007
Mean transprosthetic gradient, TPG (mmHg)	6 ± 3	13 ± 3	28 ± 11	<0.001
Mean follow-up (years)	5.1 ± 3.5	5.7 ± 3.5	5.5 ± 3.5	<0.001

Data are presented as mean ± standard deviation or number (%)

*EF* ejection fraction, *LV* left ventricular, *PPM* prosthesis–patient mismatch

### Surgical technique

All patients underwent surgery using a standard technique. After a median sternotomy, the ascending aorta and right atrium were cannulated and normothermic extracorporeal circulation with non-pulsatile flow was instituted. Myocardial protection was obtained using cold crystalloid cardioplegia (St. Thomas solution) or warm blood cardioplegia according to the surgeon’s preference. Cardioplegia was administered in an antegrade fashion through the aortic root and/or selectively in both coronary ostia to induce and maintain cardiac arrest. Retrograde administration of cardioplegia was not used. Concomitant myocardial revascularization was performed in 958 patients. Implantation of the biggest valve possible and using prosthetic valves with optimal hemodynamic profiles in patients with small annular size were strategies used to minimize the incidence of PPM. No aortic annulus enlargement techniques were used. An overview of implanted prosthetic valve types is shown in Table 2.

**Table 2:** Prosthetic valve distribution (*n* = 2,264)

Variable	Value
Prosthetic valve diameter (mm)	
Median	23 mm
Prosthetic valve type	
Mechanical	1,067 (47.1)
St. Jude Medical Standard	577 (25.5)
ATS	377 (16.7)
St. Jude Medical Regent	77 (3.4)
St. Jude Medical HP	36 (1.6)
Biological (stented)	1,197 (52.9)
Carpentier–Edwards Magna	142 (6.3)
Carpentier-Edwards Perimount	412 (18.2)
Sorin Mitroflow	291 (12.9)
Medtronic Mosaic	98 (4.3)
St. Jude Trifecta	87 (3.8)
St. Jude Medical Epic	167 (7.4)

Data are number of patients (%)

### Prosthesis–patient mismatch (PPM)

PPM was expressed by the EOAI. The EOAI was calculated by dividing the corresponding EOA of each valve type and size (registered in vitro values published by each manufacturer) by each patient’s BSA [1, 6]. PPM was defined as EOAI ≤0.85 cm^2^/m^2^ and severe PPM as EOAI <0.65 cm^2^/m^2^ [1, 3, 4]. There was no significant difference in the prevalence of PPM between patients who died within 6 weeks postoperatively (*n* = 57) and the final study population (*n* = 2,264) (10.5 vs 10.6 %, respectively; *p* = 0.994). There were no cases of severe PPM within the early deaths.

### Echocardiographic follow-up

All patients underwent postoperative transthoracic echocardiography (TTE) evaluation of the mean aortic valve pressure gradient 6 weeks after surgery. Mean pressure gradients were calculated using the modified Bernoulli equation with correction for subvalvular velocities. Two cardiologists, who have a long experience in echocardiography, supervised these measurements.

### Follow-up and late mortality

Follow-up data concerning mortality were gathered using the databases of the civil registry. The remaining data that could not be retrieved from these databases were obtained by contacting patients’ general practitioners. Twelve patients were lost to follow-up; mean follow-up was 5.5 ± 3.5 years (range 0.1–14.7 years). Patients lost to follow-up were excluded from our analysis. Late mortality was defined as all-cause death occurring later than 6 weeks after surgery.

### Statistics

Continuous variables were expressed as mean ± SD. Categorical variables were expressed as percentages. Mean values were compared by using one-way ANOVA or its non-parametric alternative, the Kruskal–Wallis test, for continuous variables and Pearson’s Chi-squared test for categorical variables.

Cumulative probability values of survival were estimated with Kaplan–Meier method and compared between groups using log-rank test.

Cox proportional-hazard regression analysis was used to determine univariate predictors and multivariate independent predictors of late mortality. Hazard ratios (HR) were reported with 95 % confidence intervals (CI). Variables considered as potential predictors for multivariable modeling were selected by univariate analyses (*p* < 0.05) and were subsequently selected by stepwise forward selection, with entry and retention in the model set at a significance level of 0.05. Goodness of fit of the final model was assessed with the Chi-squared goodness-of-fit test.

All calculations were performed using a commercially available statistical package (SPSS 20.0; SPSS Inc., Chicago, IL). Statistically significant differences were established at *p* < 0.050.

## Results

### Characteristics of the patient population

Patient characteristics are shown in Table 1. There were significantly more patients with endocarditis, a higher body weight, a higher BSA and BMI, a mechanical valve, male gender, previous cardiac surgery and a higher rate of PPM and severe PPM in the high gradient group. Patients in the high gradient group had a significantly lower age, EOA and EOAI. The low-gradient group had significantly more patients with a severely impaired LV function and more patients undergoing concomitant CABG. There were no significant differences between the three groups in other comorbidities, such as hypertension, diabetes, PVD, renal dysfunction, COPD and history of CVA. There was no significant difference in additive or logistic EuroSCORES between the groups.

### Long-term survival

Mean follow-up was 5.5 years (range 0.1–14.7 years). Total follow-up was 12,405 patient-years. Long-term survival for the entire group at 1, 3, 5, and 10 years was 97, 93, 87 and 67 %, respectively.

Survival at 1, 3, 5, and 10 years was 97, 92, 85, 66 %, respectively, for the low-gradient group, 98, 94, 99 and 68 %, respectively, for the medium gradient group and 95, 92, 87, 66 %, respectively, for the high gradient group.

**Fig. 1:**
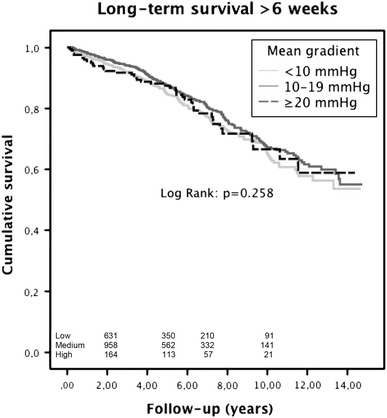
Long-term survival after transthoracic echocardiographic (TTE) evaluation of the mean transprosthetic pressure gradient (TPG) 6 weeks after surgery stratified per gradient group. Difference in survival between the groups was not significant (*p* = 0.258)

Figure 1 displays the long-term survival after transthoracic echocardiographic (TTE) evaluation of the TPG, 6 weeks after surgery stratified per gradient group. Difference in survival between the groups was not significant (*p* = 0.258).

### Predictors of late mortality

The results of Cox regression analysis for late mortality are shown in Table 3.

**Table 3:** Univariate and multivariate Cox analysis of late (>6 weeks) mortality

Variable	Univariate analysis		Multivariate analysis	
	*p*	HR (95 % CI)	*p*	HR (95 % CI)
Age (years)^a^	<0.001	1.07 (1.06–1.08)	<0.001	1.06 (1.05–1.08)
Sex (female)	0.804	1.03 (0.84–1.25)		
Severely impaired LV function (EF < 30 %)	0.040	1.57 (1.02–2.41)	0.062	1.51 (0.98–2.34)
Hypertension	0.003	1.33 (1.10–1.61)	0.449	1.08 (0.89–1.31)
Diabetes mellitus	<0.001	1.81 (1.44–2.29)	0.003	1.44 (1.13–1.83)
Endocarditis	0.115	0.62 (0.34–1.12)		
Gradient group^b^				
10–19 mmHg	0.107	0.85 (0.69–1.04)	0.781	0.97 (0.79–1.19)
≥20 mmHg	0.815	0.96 (0.69–1.34)	0.496	1.13 (0.80–1.59)
Gradient^a^	0.156	0.99 (0.98–1.00)		
Body mass index, BMI (kg/m^2^)	0.535	1.01 (0.98–1.03)		
Peripheral vascular disease (PVD)	<0.001	2.37 (1.84–3.06)	<0.001	1.81 (1.40–2.35)
Renal dysfunction	<0.001	2.21 (1.53–3.18)	0.007	1.66 (1.15–2.41)
Chronic obstructive pulmonary disease (COPD)	<0.001	1.70 (1.37–2.12)	<0.001	1.70 (1.37–2.13)
Cerebrovascular accident (CVA)	<0.001	2.09 (1.50–2.92)	0.001	1.78 (1.27–2.49)
Mechanical prosthetic valve^c^	<0.001	0.45 (0.37–0.55)	0.394	0.90 (0.70–1.15)
Concomitant coronary artery bypass grafting (CABG)	<0.001	1.56 (1.29–1.88)	0.920	0.99 (0.79–1.24)
Cardiopulmonary bypass (CPB) time (min)^a^	<0.001	1.01 (1.00–1.01)	0.011	1.00 (1.00–1.01)
Aortic cross-clamp time (min)^a^	<0.001	1.01 (1.00–1.01)	0.495	1.00 (0.99–1.00)
Prosthesis–patient mismatch (PPM) (EOAI ≤0.85 cm^2^/m^2^)	0.175	1.20 (0.92–1.56)		
Severe PPM (EOAI <0.65 cm^2^/m^2^)	0.423	1.77 (0.44–7.09)		
Previous cardiac surgery	0.151	1.29 (0.91–1.82)		

*CI* confidence interval, *EF* ejection fraction, *HR* hazard ratio, *LV* left ventricular

^a^Entered as a continuous variable

^b^Compared to low-gradient group

^c^Compared to biological valves

Univariate analysis revealed the following predictors of late mortality: age, severely impaired LV function [25], hypertension, diabetes, PVD, renal dysfunction [25], COPD, history of CVA, the use of a mechanical prosthesis, concomitant CABG, CPB time and aortic cross-clamp time. PPM, severe PPM and TPG as a continuous variable or as categorical variable (gradient group) were not significant predictors of late mortality at univariate analysis.

Multivariate analysis revealed the following independent predictors of late mortality: age, diabetes, PVD, renal dysfunction [26], COPD, history of CVA and CPB time. The use of a mechanical prosthesis, concomitant CABG, aortic cross-clamp time, and TPG were not independent predictors of late mortality at multivariate analysis. Goodness of fit of the final model was assessed with the Chi-squared goodness-of-fit test: *p* < 0.001.

## Comment

This study shows that a higher TPG measured at 6 weeks after surgery is not identified as an independent predictor of late mortality after AVR or AVR with CABG. This finding is reassuring when confronted with a postoperative pressure gradient at 6 weeks TTE follow-up.

However, we do not have follow-up data concerning the evolution of the TPG after 6 weeks. In most cases, the gradient measurement at 6 weeks will take place in a stable situation. The TPG measured in this condition will most likely be representative for the future since prosthetic-related factors, such as EOA, hemodynamic profile and surgeon-related factors such as suturing technique and sizing which may play a role in creating a TPG are already defined at that point. The etiology of TPG is complex and multifactorial and patient-related factors such as pannus [27] and thrombus formation may evolve over time. Pannus formation is a bio-reaction to the prosthesis [28–30], usually originating from the ventricular site and its structure consists mainly of myofibroblasts and an extracellular matrix such as collagen fiber [31] and thrombus can be a primary cause of pannus formation [32]. On the other hand, a TPG can induce shear stress in the peri-annular tissue, which may also contribute to pannus formation [31]. Although pannus ingrowth can occur in the late postoperative period (mean interval from previous operation 9.6 ± 2.0 years reported by Kuniyoshi et al. [33]), valve-related complications due to pannus formation are rare (incidence 0.2–4.5 % per patient year [34]) and scarcely an issue with contemporary mechanical prostheses.

Late mortality is not affected by TPG probably because the gradient measured at 6 weeks after surgery is not likely to increase significantly [35]. Zimmerli et al. [36] found that slight long-term increases in mean pressure gradients are normal findings and do not warrant a change in management strategy if unaccompanied by deterioration of symptoms or clinical signs. Postoperative TPG has to be interpreted differently than the preoperative gradient measured in patients with aortic valve stenosis, which is a progressive disease with increasing gradients over time [37]. In most cases a high TPG will still be a significant reduction in hemodynamic burden for the left ventricle compared to the even higher preoperative aortic valve gradient. This improved and stable situation for the conditioned left ventricle could be another explanation for the lack of influence of TPG on late mortality.

Although there was no significant difference in additive and logistic EuroSCORES [38, 39] and both study populations were homogeneous for most risk factors, some baseline patient characteristics were significantly different between the two groups. Patients in the high gradient group not only had a significantly higher BSA, but also a lower EOA resulting in a higher prevalence of PPM and severe PPM in this group. Nevertheless, PPM and severe PPM were not significant predictors of late mortality and therefore unlikely to have a negative effect on survival in the high gradient group. The fact that PPM does not affect long-term survival is consistent with other studies [7, 19, 24, 40–44].

Most operative characteristics, such as the use of mechanical valve prostheses, concomitant CABG, CPB time, aortic cross-clamp time were significantly different between the groups. Only CPB time was an independent predictor of late mortality whereas aortic cross-clamp time was not. Aortic cross-clamp time is a reflection of the duration of the technical repair, whereas CPB time is a reflexion of the duration of the technical repair time and the time the patient needs to wean from CPB, hence a reflexion of the general condition of the heart.

An important limitation is the retrospective design of this study. Therefore, some baseline patient characteristics were significantly different between the gradient groups. However, there was no significant difference in most comorbidities and EuroSCORES between the groups. Secondly, we focussed on the patients undergoing TTE follow-up at 6 weeks after surgery and the effect of a high TPG on late mortality, thus excluding patients that died before having their TTE follow-up at 6 weeks. The low prevalence of severe PPM (*n* = 7, 0.3 %), possibly caused by the above-mentioned surgical strategies to avoid PPM, limits the statistical analysis of this group. On the other hand, it is important to note that severe PPM is extremely rare when using straightforward surgical strategies to avoid PPM. In addition, the primary end-point was all-cause mortality. We were not able to retrieve the cause of death that might be equally important and we did not have any information about quality of life after AVR in relation to the TPG. Finally, the relatively short mean follow-up of 5.5 years also limits conclusions about the long-term effect of TPG on survival.

## Conclusions

In conclusion, our findings indicate that TPG measured at 6 weeks after AVR or AVR with CABG is not an independent predictor of all-cause late mortality and there is no significant difference in long-term survival between patients with a low, medium or high TPG.

## Abbreviations

AVR: Aortic valve replacement
BMI: Body mass index
BSA: Body surface area
CABG: Coronary artery bypass grafting
CI: Confidence interval
COPD: Chronic obstructive pulmonary disease
CPB: Cardiopulmonary bypass
CVA: Cerebrovascular accident
EF: Ejection fraction
EOA (I): Effective orifice area (index)
HR: Hazard ratio
LV: Left ventricle
PPM: Prosthesis–patient mismatch
TEE: Transesophageal echocardiography
TTE: Transthoracic echocardiography
